# Death is overrated: the potential role of detection in driving virulence evolution

**DOI:** 10.1098/rspb.2023.0117

**Published:** 2023-03-29

**Authors:** David A. Kennedy

**Affiliations:** Department of Biology, Center for Infectious Disease Dynamics, The Pennsylvania State University, University Park, PA, USA

**Keywords:** evolution of virulence, virulence–transmission trade-off, detection cost, mortality cost, pathogen evolution

## Abstract

A common assumption in the evolution of virulence theory literature is that pathogens transmit better when they exploit their host more heavily, but by doing so, they impose a greater risk of killing their host, thus truncating infectious periods and reducing their own opportunities for transmission. Here, I derive an equation for the magnitude of this cost in terms of the infection fatality rate, and in doing so, I show that there are many cases where mortality costs are too small to plausibly constrain increases in host exploitation by pathogens. I propose that pathogen evolution may often be constrained by detection costs, whereby hosts alter their behaviour when infection is detectable, and thus reduce pathogen opportunities for onward transmission. I then derive an inequality to illustrate when mortality costs or detection costs impose stronger constraints on pathogen evolution, and I use empirical data from the literature to demonstrate that detection costs are frequently large in both human and animal populations. Finally, I give examples of how evolutionary predictions can change depending on whether costs of host exploitation are borne out through mortality or detection.

## 1. Introduction

The seminal works of Anderson & May [1,2] changed the way that biologists thought about the evolution of pathogen virulence, defined as the severity of disease signs or symptoms caused by infection with a particular pathogen. Before Anderson & May, the conventional wisdom was that pathogens would evolve to be avirulent over time [3], since a highly virulent pathogen risks killing its host and by killing its host a pathogen truncates its own infectious period and reduces its own fitness. Anderson & May articulated that natural selection favours pathogen variants that maximize their own fitness. If virulence were correlated with other epidemiological parameters such as infectiousness or time to recovery, intermediate levels of virulence could maximize fitness, and thus be evolutionarily adaptive. The idea they proposed, ‘trade-off theory’, is that the cost of virulence, which they assumed was a truncated duration of infectiousness caused by host mortality, trades off against other benefits such as an increased rate of transmission or a decreased rate of recovery. This work has been hugely influential, and the trade-off theory that they proposed has since been termed the ‘new conventional wisdom’ [4]. Ultimately, trade-off theory was meant to explain why evolution has generated pathogens that have intermediate levels of virulence. That is, (i) why do pathogens harm their hosts at all, and (ii) why do they not harm their hosts more?

There are only three sets of explanations for why evolution has allowed pathogens to maintain virulence. Either there is no genetic variation for reduced virulence, selection is too weak to eliminate virulence, or virulence is associated with some direct or indirect fitness benefit to the pathogen [3]. Although it is possible to point to specific examples where each of these explanations apply, the widespread detection of variation in virulence (e.g. [5]) and the observation that virulence often increases during serial passage experiments [6] challenge the generality of these first two explanations. By contrast, a recent meta-analysis of experimental studies on virulence evolution found that within pathogen species, replication rates within hosts positively correlate with both transmission potential and virulence [7], lending support to the third explanation. It has thus been generally accepted that for most pathogens some degree of virulence provides or is correlated with a fitness benefit in some environment where selection is acting.

**Figure 1.**
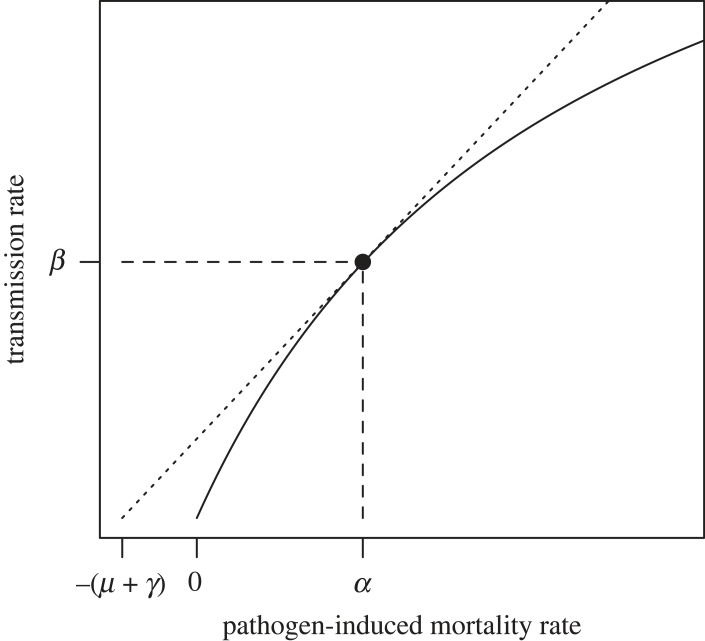
Classical formulation of the virulence–transmission trade-off. The solid curve shows a possible trade-off between transmission rate and pathogen-induced host mortality rate. The evolutionarily optimal values of transmission rate *β* and mortality rate *α* according to original theory are depicted by the point where the dotted line touches the curve [4]. This optimal value depends on the baseline host mortality rate *μ* and the infection recovery rate *γ*.

So if virulence is associated with fitness benefits, why are pathogens not more harmful to their hosts? Classical trade-off theory proposes that pathogens are not more harmful to their hosts because the fitness benefits associated with increased virulence saturate relative to the fitness costs of increased virulence [4]. Translated to a mathematical framework, the typical assumption is that increases in transmission rate saturate relative to increases in host mortality (figure 1; see [8] for a formal derivation). Such a relationship may emerge due to within host processes [9], and has been seen in some biological systems (e.g. [10,11]), but this saturation has not been generally detected [7]. In fact, experimental data in support of trade-off theory has been restricted to a relatively small number of studies and systems [3,4,12–14], leading to questions about the usefulness of trade-off theory entirely [13,15,16]. Rather than disregard trade-off theory, some have argued that more robust experimental support for trade-off theory has been lacking due to difficulties in designing appropriate experiments [3] and in collecting suitable proxies for virulence costs and transmission benefits [14]. Here, I argue that an additional reason few experiments have found evidence supporting trade-off theory may be because these experiments assume the cost of virulence is borne out through a reduction in the duration of infections due to host mortality, despite the fact that mortality costs are often be too small to plausibly be the factor constraining virulence evolution.

Behaviour and behavioural changes are being increasingly recognized as important drivers of infectious disease dynamics in humans [17] and other animals [18]. Changes in behaviour alone are capable of tipping the balance from localized pathogen extinction to successful disease emergence [19,20]. It thus follows that infection-induced changes in host behaviour could impose substantial pressure on pathogen evolution. For both human and animal diseases, host contact rates often substantially decline upon symptomatic infection [21–26]. In one case, a positive correlation was even documented within a single pathogen between disease severity and the change in contact rates [27]. Yet with few exceptions (e.g. [28,29]), the role of behaviour on virulence evolution has been largely neglected in favour of a focus on mortality costs. The critical role of behaviour in driving disease ecology could lead one to wonder whether changes in behaviour following infection may nevertheless be a key evolutionary force shaping pathogen virulence.

I argue that the cost of virulence can often play out through morbidity-induced reductions in contact rates—which I refer to as a ‘detection cost of virulence’—and that this cost of virulence can often greatly outweigh the cost of infection-induced mortality. Therefore, assuming a positive correlation between mortality and sublethal disease severity, pathogen virulence may frequently be constrained by detection costs rather than mortality costs. In addition to being supported by the empirical literature, as I will discuss, this argument builds on several previously published concepts. Ewald [28,29] long ago proposed a trade-off between virulence and transmission mode that implicitly included a virulence-detection trade-off. This idea was later formalized [30,31], and the concept has been discussed by many others (e.g. [3,27]). Likewise, Ebert & Bull [15] and Bull & Lauring [16] previously discussed that virulence, in the context of mortality, is likely to impose only an indirect and weak evolutionary cost.

Here, I show that, under the assumptions of a mortality-rate–transmission-rate trade-off, the cost of virulence can be written in terms of the infection fatality rate (defined as the fraction of all infections that result in disease-induced host death). Using this new form, I show that in contrast to detection costs, mortality costs are often too weak to constrain pathogen evolution, particularly in human diseases where even a 1% infection fatality rate is often considered large.

## 2. Model and results

### (a) The cost of mortality

The original formulation of the virulence–transmission trade-off arises from analysis of a classic SIR model based on the models of Anderson & May [32].

2.1 $$\frac{dS}{dt}=r(1-N)+\phi R-\beta SI-\mu S,$$

2.2 $$\frac{dI}{dt}=\beta SI-\alpha I-\gamma I-\mu I$$

2.3 $$\mathrm{and}\;\frac{dR}{dt}=\gamma I-\phi R-\mu R.$$

Above, *S*, *I* and *R* are the respective densities of susceptible, infectious and recovered hosts. *N* is the total population density derived by summing *S*, *I* and *R*. *r* is the maximum *per capita* birth rate, *ϕ* is that rate at which immunity wanes, *β* is the transmission rate, *γ* is the recovery rate, *μ* is the baseline host mortality rate and *α* is the pathogen-induced host mortality rate.

Under the assumptions of this model and any model that excludes nonlinear environmental feedbacks such as spatial structure [33,34], coinfection [8,35], superinfection [36], host heterogeneity [37] and nonlinear transmission [38], a pathogen strain that maximizes the basic reproductive number *R*_0_ will competitively exclude all other pathogen strains once the system reaches an equilibrium [8]. As explained in [38], this is because under these assumptions, the reproductive number includes only a single dimension of environmental feedback and so adaptive dynamics yield the same evolutionarily stable strategy as *R*_0_ maximization. It thus follows that natural selection will lead to the evolution of a pathogen strain that maximizes *R*_0_ [1].

In the above model, the basic reproductive number is

2.4 $$R_{0}=\frac{\beta N}{\alpha +\gamma +\mu }.$$

This formulation of *R*_0_ illustrates the paradox of virulence pointed out previously by May & Anderson [2]. That is, all else equal, a strain with lower virulence (i.e. smaller *α*) would have a higher *R*_0_, and thus, pathogens should evolve to be avirulent. However, if transmission rate *β* or recovery rate *γ* were functions of virulence, it need not be the case that low virulence is always favoured. Famously, *R*_0_ can be maximized at intermediate virulence if the transmission rate *β* is a saturating function of the pathogen-induced mortality rate *α* (figure 1). This so-called virulence–transmission trade-off is by far the most widely invoked explanation for the maintenance of virulence in nature.

According to the principle of *R*_0_ maximization, a new pathogen variant would be able to invade and displace an existing pathogen strain provided the new value of *R*_0_ is greater than the old value of *R*_0_. Under the assumption that recovery rate *γ* is the same for the two pathogen variants, this can be reduced to (electronic supplementary material, S1.1)

2.5 $$\frac{\Delta \alpha }{\alpha_{m}+\gamma +\mu }<\frac{\Delta \beta }{\beta_{m}}.$$

Above, I use the symbol Δ as shorthand for the difference between the old and new values for a parameter, such that Δ*X* corresponds to *X*_*m*_ − *X*_*o*_, where subscript *m* denotes the mutant variant and subscript *o* denotes the original variant. Inequality 2.5 leads to the well-known result that if the transmission rate *β* is a saturating function of disease-induced mortality rate *α*, then an optimal level of virulence can be derived as shown in figure 1.

I note that there is frequently confusion regarding the parameter *α*, since rate parameters can be difficult to interpret. This parameter is the per unit time risk of disease-induced death given that a host is still alive and has not yet recovered from infection, which is distinct from the arguably more intuitive infection fatality rate *F* (or the fraction of all infections that result in disease-induced death). However, these two values are related such that in a classical SIR model like that described by equations (2.1)–(2.3), *F* = *α*/(*α* + *γ* + *μ*). Under the assumption that recovery rates do not differ between variants [39], Inequality 2.5 can be rewritten in terms of *F*, leading to the conclusion that a new mutation will spread if (electronic supplementary material, S1.2)

2.6 $$\frac{\Delta F}{1-F_{o}}<\frac{\Delta \beta }{\beta_{m}}.$$

Here, *F*_*o*_ is the infection fatality rate of the original variant, and *β*_*m*_ is the transmission rate of the mutant variant. The above inequality thus states that a new variant will be able to invade and displace the current pathogen if the percentage decrease in infection survival rate 1 − *F*, is less than the percentage increase in the transmission rate *β*. The left side of this inequality can therefore be viewed as the costs of virulence and the right side can be viewed as the benefits. Note that inequality (2.6) and all the inequalities in this paper are valid regardless of whether virulence and transmission rate are related. The inequality merely determines which of two variants would be selectively favoured, not how likely it is for such variants to arise in the first place.

The advantage of inequality (2.6) over the standard formulation (inequality (2.5)) is that it shows that the cost of mortality depends on per cent changes in survival rather than per cent changes in mortality. Perhaps non-intuitively, this means that the same change in the infection fatality rate produces a large fitness cost for pathogens with initially high mortality (i.e. *F*_*o*_ ≈ 1) but a small fitness cost for pathogens with initially low mortality (i.e. *F*_*o*_ ≈ 0). The consequence of this asymmetry means that for pathogens with initially low infection fatality rates, variants with increases in virulence should be able to invade and spread provided the per cent increase in the transmission rate *β* is greater than the absolute change in the infection fatality rate *F* (inequality (2.6)). Put another way, if a pathogen had an infection fatality rate *F* = 1/1000 (something akin to an influenza A virus in humans), the infection fatality rate would have to increase by at least 10-fold to prevent the spread of a variant that increased transmission by merely 1%. The fact that there are pathogens with extremely low virulence, however, suggests that something other than host mortality must be constraining the virulence of these pathogens, since virtually any mutation that increased transmission would be selectively advantageous in these systems. To clarify the term ‘extremely low virulence’ it is helpful to quantify the total cost of virulence by calculating the per cent that *R*_0_ is reduced by mortality relative to a fully avirulent variant. This value is equal to *F* (electronic supplementary material, S1.3). My use of the term extremely low virulence is meant to describe pathogens in which this value is small (e.g. *F* < 0.05), even though many would not consider a human pathogen that kills 5% of hosts to have low virulence, let alone extremely low virulence.

To further illustrate this point that mortality costs are often small, consider a theoretical pathogen with a low infection fatality rate *F*_*o*_ ≈ 0, something akin to a rhinovirus that causes the common cold. If this pathogen has an *R*_0_ of 5 and an infection duration of 5 days, then that implies each infection produces 1 new infection per day. Inequality (2.6) tells us that a mutation that increased its per day infectiousness from 1.00 to 1.01 would be evolutionarily favoured even if the mutation increased the infection fatality rate from 0% to approximately 1%. Notably, a 1% change in transmission is small relative to differences in transmission rates typically detected between field isolates of pathogens and parasites (e.g. [40]), but this change in mortality rate is larger than the difference between a common-cold-causing rhinovirus and SARS-CoV-2 [41]. Theory thus predicts that if the main cost of virulence were host mortality, the common cold could become as severe as COVID-19 if such an increase in virulence also provided just a 1% increase in the transmission rate of the virus. Yet no such variant has ever spread, and there has never even been a documented cluster of rhinovirus infections with COVID-like mortality rates. Similarly, an increase in transmission rate from 1.00 to 1.10 could justify an infection fatality rate as high as 9%, which is comparable to the infection fatality rate of the 2003 SARS virus [42]. Nearly identical numbers can be derived for pathogens and parasites that are typically thought of as less mild, such as influenza A viruses, measles virus, *Plasmodium falciparum* and SARS-CoV-2. It is therefore highly implausible to conclude that mortality costs constrain increases in the transmissibility of these pathogens, unless we also believe that transmissiblity has very little potential to evolve.

**Figure 2.**
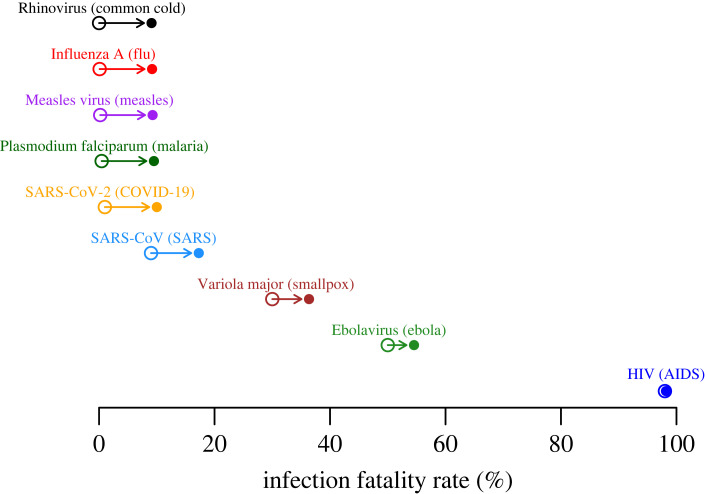
Under the assumption that host mortality constrains virulence, moderate changes in transmission rates can justify large increases in the infection fatality rate. Open circles indicate approximate infection fatality rates for various pathogens and parasites (values are for illustration purposes and may not be exact). Under the assumption that mortality costs constrain evolution, filled circles indicate the maximum infection fatality rate that would be evolutionarily favoured if it were accompanied by a 10% increase in the transmission rate (Δ*β* / *β*_*o*_). Note that the differences in severity are nearly indistinguishable on this scale between classically mild pathogens such as a rhinovirus that causes the common cold and pathogens and parasites more often considered to be severe such as SARS-CoV-2 or *Plasmodium falciparum*. Regardless, the illustrated 10% increase in transmission is enough to justify an otherwise harmless rhinovirus evolving to become approximately 10-fold more lethal than SARS-CoV-2 or *Plasmodium falciparum*.

Figure 2 illustrates how the impact of mortality costs change as a function of the infection fatality rate. Note that the magnitude of maximum change in the infection fatality rate (i.e. length of the arrows) that would be selectively favoured drastically shrinks as the initial infection fatality rate gets large. It is also worth pointing out that this figure is independent of the underlying shape of any trade-off curve between transmission and host mortality that may exist since the figure illustrates the maximum tolerable trade-off. A trade-off curve would merely be used to determine whether there are accessible variants that fall within the adaptive range.

**Figure 3.**
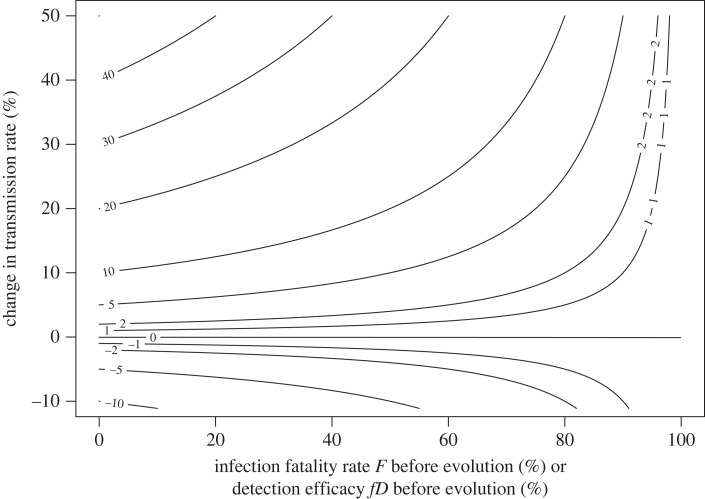
Contour lines show the maximum absolute change in the infection fatality rate or detection efficacy that would be evolutionarily favoured for a given per cent change in transmission rate (i.e. Δ*β*/*β*_*m*_). Note that when the original infection fatality rate is small (i.e. small values on *x*-axis) any absolute change in the infection fatality rate can be fully balanced by an equivalent percentage change in the transmission rate. The horizontal nature of the contour lines at small to moderate *x*-axis values indicates that costs of mortality are small unless the original infection fatality rate is large. Thus it is only when infection fatality rates before evolution are large, that increases in infection fatality pose a strong constraint on pathogen evolution.

For the above theory, I have thus shown that the magnitude of a mortality cost increases as either of two factors increases: the change in the infection fatality rate Δ*F*, or the baseline infection fatality rate *F*_*o*_. Mortality costs can thus strongly constrain further increases in mortality for pathogens that begin with extremely high infection fatality rates *F* (figure 3) such as for lethal, chronic infections like human immunodeficiency virus (HIV). Notably, pathogens with extremely high infection fatality rates are the very ones in which trade-offs between mortality costs and transmission benefits have been shown to be potential drivers of evolution [11,43,44]. However, trade-offs between virulence and transmission would have to be quite steep for pathogens with low or moderate infection fatality rates to constrain pathogen evolution (figure 2). One is therefore left to wonder what might constrain virulence in other systems.

### (b) The cost of detection

While there are many possible ways that costs of virulence may arise, I propose that behavioural change due to the detection of infection may be a common factor that constrains virulence evolution. For instance, if someone realizes they have symptoms of an infection, they may self-isolate thereby reducing transmission opportunities. If they do not self-isolate, but they appear ill to others, they may be avoided. Alternatively, they may simply feel too sick to conduct their normal daily activities again reducing transmission opportunities through reduced contact with conspecifics. Regardless of the mechanism, however, causing detectable infection could negatively impact a pathogen’s own fitness, and presumably moreso for increasingly severe disease. Notably, such behavioural change need not be unique to humans.

To formalize this concept, consider an alternative SIR-type model

2.7 $$\frac{dS}{dt}=r(1-N)+\phi R-\beta SI_{n}-(1-f)\beta SI_{d}-\mu S,$$

2.8 $$\frac{dI_{n}}{dt}=(1-D)(\beta SI_{n}+(1-f)\beta SI_{d})-\gamma I_{n}-\alpha I_{n}-\mu I_{n},$$

2.9 $$\frac{dI_{d}}{dt}=D(\beta SI_{n}+(1-f)\beta SI_{d})-\gamma I_{d}-\alpha I_{d}-\mu I_{d}$$

2.10 $$\mathrm{and}\;\frac{dR}{dt}=\gamma I_{n}+\gamma I_{d}-\phi R-\mu R.$$

The above model is identical to the one before except that the infected class has been split up into two groups *I*_*n*_ and *I*_*d*_, respectively, describing the not detected and detected infections. *D* is the fraction of new infections that are detected, and *f* is the reduction in transmission that occurs in detected infections relative to non-detected infections. Note that these new parameters *f* and *D* are fractions rather than rates and are thus bounded between 0 and 1. In my text below, I assume that there is a positive correlation between the transmission rate *β,* and the likelihood of detection *D* and altered behaviour *f* similarly to the previously assumed positive correlation between transmission rate and mortality. Presumably, the parameters *D* and *f* will also be correlated with disease-induced mortality *α* in many biological systems. Note that none of the math that follows requires that there be correlations between any of these parameters, but the conclusion that mortality is often constrained by detection costs does rely upon a positive correlation between mortality and detection.

In electronic supplementary material, S1.4, we derive the basic reproductive number,

2.11 $$R_{0}=\frac{\beta N(1-fD)}{\alpha +\gamma +\mu }.$$

As in the case of the SIR model described by equations (2.1)–(2.3), the reproductive number scales linearly with the population size of susceptible hosts (*N* in the context of *R*_0_, and *S* in the context of the effective reproductive number). Note that no additional environmental feedbacks have been introduced relative to the more classical model described by equations (2.1)–(2.3), and thus *R*_0_ maximization can again be used to identify the evolutionarily stable strategy [38]. The above formulation of *R*_0_ illustrates the cost of detection. Specifically, the absolute fitness of a pathogen (defined by the reproductive number) is reduced by *fD* per cent due to the detection of infection. Note that this parameter combination describes the fraction of new infections that are prevented because of pathogen detection. This may be realized through, for example, a reduction in contact rate or a reduction in infectiousness given contact. More highly virulent pathogens presumably lead to detection in a larger fraction of hosts (i.e. increased *D*), and more stringent actions to reduce transmission once detected (i.e. increased *f*) [27]. The net effect of this change in behaviour is to decrease overall transmission opportunities and thus *R*_0_. If we assume that increased disease severity correlates with increased transmission potential in the absence of detection *β* but increased detection costs *fD*, then it is possible for *R*_0_ to be maximized at intermediate levels. One might also reasonably expect a positive relationship between detection costs and the infection fatality rate *F*, but this relationship may be highly nonlinear.

As before, a new pathogen variant would be able to displace an existing pathogen if the new value of *R*_0_ is greater than the old value of *R*_0_. As with the mortality-cost-only model, I assume the recovery rate *γ* is unchanged by the evolution of virulence (i.e. no trade-off between virulence and recovery). In electronic supplementary material, S1.5, I show that this means a new variant will be capable of invading if

2.12 $$\frac{1-f_{m}D_{m}}{1-f_{o}D_{o}}\;\frac{\Delta F}{1-F_{o}}+\frac{\Delta (fD)}{1-f_{o}D_{o}}<\frac{\Delta \beta }{\beta_{m}}.$$

Above, Δ(*fD*) is the change in transmission caused by the detection of infections and it is defined as *f*_*m*_*D*_*m*_ − *f*_*o*_*D*_*o*_. As in inequality (2.6), the left-hand side of Inequality (2.13) can be conceptualized as the costs of virulence and the right-hand side can be conceptualized as the benefits. A new variant would be selectively favoured when the costs are smaller than the benefits. Note that in contrast to before, the left-hand side of the above inequality depends on both mortality and detection. Perhaps unsurprisingly, this inequality demonstrates that either mortality costs or detection costs could in principle constrain virulence evolution, and that they could even combine together to generate such a constraint.

To help conceptualize these costs, consider a mutation that impacts only mortality or only detection. By definition, if a mutation impacts only mortality, then *f*_*m*_*D*_*m*_ = *f*_*o*_*D*_*o*_. In this case, we exactly recover Inequality (2.6), and thus we recover all the same conclusions regarding the size of the mortality cost. Alternatively, consider a mutation that impacts only detection costs, meaning *F*_*m*_ = *F*_*o*_. In this case, we arrive at

2.13 $$\frac{\Delta (fD)}{1-f_{o}D_{o}}<\frac{\Delta \beta }{\beta_{m}}.$$

Note the similarities to inequality (2.6). Here, the magnitude of the detection cost scales with the per cent change in ineffective interventions, analogously to how the mortality cost scales with the per cent change in survival. Nevertheless, a key difference emerges between inequalities (2.6) and (2.13) when one considers that the parameter *F* describes the fraction of infections that result in disease-induced death, whereas the parameter combination *fD* describes the fractional reduction in transmission caused by the detection of infection. In humans, a disease that on average causes mortality in one per cent of infected hosts would be considered highly virulent, whereas a disease that causes an infected individual to contact on average one per cent fewer individuals (perhaps by having a 1% chance of missing school or work) would be considered fairly mild.

I previously showed using an example that host mortality is unlikely to constrain the evolution of pathogen virulence for pathogens with initially low infection fatality rates *F*_*o*_. Again consider the same hypothetical pathogen with an infection duration of 5 days and an *R*_0_ of 5, but this time, focus on the cost of detection. Assume that an individual becomes less likely to attend school, work, or otherwise contact conspecifics with increasingly severe symptoms, and that the vast majority of transmission occurs during these activities. Using this information, we can ask under what circumstances a new variant that causes the average infected host to stay home 1 day would be able to displace a less severe variant that causes the average host to not stay home at all (i.e. virtually no initial cost of virulence as in the mortality cost example above). Using the above details, we can calculate the key parameters: *f*_*m*_*D*_*m*_ = 1/5, *f*_*o*_*D*_*o*_ = 0. Plugging these values into inequality (2.13) leads to the conclusion that this variant would only be able to invade if it were accompanied by at least a 20% increase in transmission. This can be visualized in figure 3.

If we were to relate this example with detection costs to the previous example with mortality costs, transitioning from a 0% infection fatality rate to a 1% infection fatality rate is an equivalent cost to transitioning from a 0% to 1% chance of staying home due to infection given that you are infected. These changes in the pathogen would be selectively favoured if they led to an increase in transmission of just 1% or more (inequalities (2.6) and (2.13)). While an increase in mortality to 1% would almost certainly be documented if it were to evolve in human populations, an increase in the absence rate to 1% on days in which an individual is infected almost certainly would not. Notably, the average chance of staying home given infection is probably many times greater than 1% for many pathogens, given that the average American misses more than 2% of all work days for health-related reasons [45]. As with mortality costs, when detection costs are initially higher (as *f*_*o*_*D*_*o*_ gets closer to 1) the same size change in detection generates an even stronger constraint on pathogen evolution (inequality (2.13)).

Presumably many infectious diseases, including non-human diseases, could be constrained by costs of detection. However, detection would not have much impact on limiting disease severity if reductions in transmission were small following the detection of infection (i.e. *f* is small), if a very small fraction of infections were detected (i.e. *D* is small), or if large fractions of the infectious period occurred prior to the time when detection would be possible (although not captured by this model). Likewise, host-induced mortality can be a strong constraint on virulence evolution if the infection fatality rate *F* is large. To determine whether virulence is more strongly shaped by a mortality–transmission trade-off or a detection-transmission trade-off, one can combine inequalities (2.6) and (2.13) to ask

2.14 $$\frac{\Delta F}{1-F_{o}}\overset{?}{>}\frac{\Delta (fD)}{1-f_{o}D_{o}}.$$

**Figure 4.**
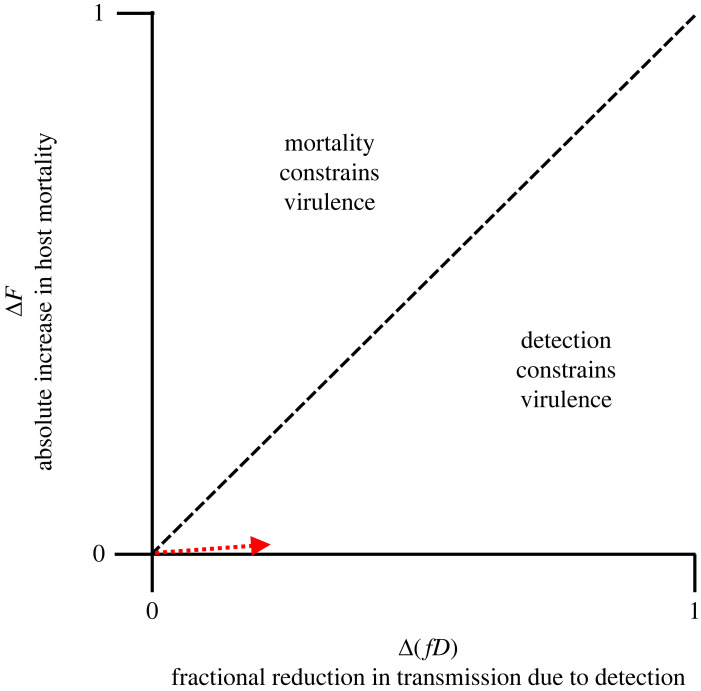
A graphical representation of Inequality (2.14) for a pathogen with initially low virulence. The cost from an *x*% reduction in average transmission is equivalent to the cost from an *x*% increase in the infection fatality rate. The dashed line is the 1:1 line. Above the dashed line, virulence is constrained by mortality costs, and below it, detection costs. The dotted red arrow depicts the example provided in the main text of a pathogen with initially low virulence that evolves higher virulence in the form of either killing 1% of infected hosts (i.e. roughly equivalent to SARS-CoV-2 infection) or causing infected hosts to stay home for one day (roughly equivalent to infection with a virus that causes the flu or the common cold). Notably, the cost of the former is much smaller than the cost of the latter despite the fact that most would consider the former more virulent than the latter.

When the left-hand side of the above expression is larger than the right-hand side, mortality will impose a stronger constraint on virulence evolution than detection, and vice versa. For a pathogen with low virulence, the denominators on both sides are close to one meaning that we can visualize this inequality using only the numerators (figure 4). This demonstrates that for pathogens with relatively low virulence, detection will generally be a stronger constraint on virulence evolution than mortality, since presumably all individuals that die change their behaviour whereas not all individuals that change their behaviour die. It is, however, worth noting that this conclusion depends on the precise functional form that connects transmission rate, infection fatality rate, and detection, and these functional forms can only be determined through empirical work.

## 3. Discussion

The new conventional wisdom states that pathogens evolve to balance the costs and benefits of virulence and its associated traits. Typically, the cost of virulence is assumed to be a truncated infectious period due to disease-induced host mortality. Here, I have argued that this cost is often too weak to constrain virulence. To do this, I have rewritten the virulence–transmission trade-off equation in terms of infection fatality rate (i.e. the fraction of infections that result in host death *F*) rather than in terms of the per day infection-induced death rate (i.e. *α*). This formulation makes explicit that mortality-based evolution of virulence theory predicts that a novel variant would be able to displace an existing pathogen variant if the per cent decrease in host survival is less than the per cent increase in the rate of transmission (inequality (2.6)). This inequality thus states that mortality costs are small for all but the most virulent pathogens (figures 2 and 3). While it is possible for a small cost to nevertheless constrain pathogen evolution, I propose that behavioural changes that result from the detection of infection may be a more common constraint on virulence evolution. Using a modified SIR model that explicitly allows for costs of detection, I show that detection costs can be quite large, even for pathogens that might be considered mild, and therefore detection may often be a much stronger constraint on virulence evolution than host-induced mortality (figure 4).

Virulence has been defined differently by different researchers [3,14,46,47]. For example, virulence can be defined as the pathogen-induced reduction in host fitness [11], as the per day pathogen-induced host mortality rate [1], as the fraction of hosts that die from infection [39], or in numerous other ways [14,47], and these differences can lead to fundamentally different conclusions [39]. Here, I have defined virulence as the severity of disease signs or symptoms caused by infection with a pathogen. The argument that I have put forward applies to this definition of virulence specifically. While it may apply to other definitions of virulence as well, this application relies on correlations in ‘virulence scores’ between the definitions.

It has previously been noted that costs of mortality are small [15,16]. However, since the classical SIR formulation relies on per day rates of mortality given infection, which can only be interpreted in context with other rates, it has been difficult to intuit precisely how small mortality costs are. I provide an analytical expression (inequality 2.6). This expression states that when infection fatality rates are small, a per cent change in transmission rate can balance an equivalent absolute change in the infection fatality rate (figures 2 and 3). Note that this expression, regardless of how virulence and transmission rate are related, can be used to determine which of two variants would be selectively favoured. Under the assumption that mortality limits disease severity, a 1% increase in transmission can thus justify something as harmless as a virus that causes the common cold evolving to become something as deadly as SARS-CoV-2, yet a common cold virus has never evolved to be so deadly. Certainly, other factors must constrain virulence in such systems. Previous work has considered alternative factors such as changes in host recovery rates [48], but evidence supporting such costs has been generally restricted to a small set of systems [49]. Here, I have argued that changes in host behaviour following infection may constrain virulence evolution in many systems.

Host behaviour is widely recognized to influence infectious disease dynamics [17,18], but recognition of its potential to drive evolution of virulence has received less attention. It only stands to reason that when infection-induced behavioural changes affect opportunities for onward transmission, and disease severity alters the degree of behavioural change, there will be opportunities for natural selection to shape disease severity. This idea was originally proposed by Ewald [28,29] to argue that vector transmitted diseases should evolve to be more virulent than directly transmitted diseases since they do not rely on their hosts for dispersal. However, definitive data supporting Ewald’s argument regarding transmission mode are still lacking [50]. While a disconnect between his assumptions and his conclusions may be due to variation in system specific details [31], support for mortality costs in the case of myxomatosis [43,51] led to mortality being viewed as a reasonable constraint on virulence evolution. Myxoma virus, notably however, has extremely high infection fatality rates. While myxomatosis and others systems with high infection fatality rates can generate extremely large mortality costs, many other systems have low or moderate infection fatality rates. I have shown that when infection fatality rates are low, mortality is not a reasonable constraint on virulence evolution. This result may help to explain why surprisingly few data support mortality as a constraint on pathogen evolution [4,7,12–16].

At some level, this conclusion may be obvious. Both mortality and detection could in principle constrain virulence evolution provided they truncate infectious periods or otherwise reduce opportunities for transmission (Inequality (2.12)), but, at least in the case of human pathogens, death tends to be a rare outcome of infection whereas detection tends to be a common outcome. It thus follows that detection costs may often be larger than mortality costs. It is worth stressing that the precise magnitude of these costs and their size relative to each other will depend on the relationship between mortality, detection and transmission. Unfortunately, these relationships have not been well characterized. That said, by delving into specific studies we can investigate the typical magnitude of detection costs.

Few data are yet available to quantify precisely how large detection costs are, but the data that do exist suggest these costs can be quite large. A study of influenza-like-illness (ILI) during the 2009 influenza pandemic found that people with ILI reduced their per day contacts by 75% (implying *f* ≈ 0.75), and the average duration of contact also declined [23]. Despite this reduction, two-thirds of transmission was attributable to symptomatic infection, suggesting a steep trade-off between contact rate and infectiousness given contact [23]. Another study using seasonal influenza documented a negative correlation between morbidity scores and activity levels among people with detected infections, and even proposed that this reduction in activity may pose a constraint on virulence evolution in that system [27]. Similarly, a survey study on behavioural change following diagnosis with various sexually transmitted diseases reported that 71% (*D* < 0.71) of men modified their behaviour in ways that would reduce opportunities for disease transmission (e.g. increased condom use, reduced frequency of sex) [21]. Isolation and quarantine following the detection of SARS-CoV-2 infection in an individual or in a close contact of an individual likewise is thought to have large impacts on disease transmission [52]. Nevertheless, more data are needed to establish whether the magnitude of these detection costs are typical for human diseases.

Similar magnitude effects are seen in the animal world. When wild house mice were experimentally injected with lipopolysaccharide (LPS) to induce disease symptoms, 40% of the mice disconnected entirely from their social groups [24]. Although these mice did not have an infectious disease, the change in behaviour brought on by a general immune response would have substantially reduced opportunities for pathogen transmission if it were brought on by a pathogen (*D* = 0.4 and *f* = 1) [24]. Vampire bats injected with LPS also showed large changes in behaviour, with 85% less time spent grooming conspecifics and 19% less time spend being groomed by conspecifics (implying 0.19 < *fD* < 0.85) [26]. Analogous patterns were found in guppies infected with an ectoparasite. Guppies typically form groups called shoals, but when infected guppies were added to otherwise healthy populations, the healthy fish actively avoided the infected guppies causing fission events at twice the rate of controls, and associations when they did occur were half as long in duration (implying *fD* > 0.5) [22]. In a eusocial ant species, when colony workers were experimentally infected with a fungal pathogen, the social network of the colony changed in ways that reduced opportunities for disease transmission, including a shift such that experimentally infected worker ants spent 20% more time outside of the nest than controls (implying *fD* ≈ 0.20) [25]. As can be seen from Inequalities (2.6) and (2.13), mortality and detection produce costs of identical magnitude when *F* = *fD*, and so the detection costs described here and in the human examples are as large as mortality costs that would be imposed by pathogens with fatality rates between approximately 20% and 80%. While there may be some pathogens capable of such high mortality rates, particularly for insect diseases and some of the more harmful wildlife diseases, there are likely numerous others for which detection costs will drastically outweigh mortality costs.

Notably, detection costs may even be playing a role in limiting virulence for some of the systems where virulence–transmission trade-offs have been best documented. For example, in *Mycoplasma gallisepticum* where prior immune history enhances the spread of highly virulent strains [53], interaction rates between birds are approximately 15% lower for infected birds than non-infected birds (*fD* = 0.15) [54]. Likewise, for monarch butterflies infected with the parasite *Ophryocystis elektroscirrha* at high spore loads, reductions in mating success that prevent transmission to offspring actually impose a larger fitness cost to the parasite than mortality, captured as pupal emergence [10], although perhaps not significantly so (0.1 ≤ *F* ≤ 0.5 versus 0.3 ≤ *fD* ≤ 0.6 at high spore loads).

The above examples demonstrate that detection costs, when quantified, have tended to be large, equivalent to mortality costs imposed by pathogens that kill about 20–80% of infected hosts. While the above data may be subject to some of the same publication biases that have previously plagued trade-off theory [7], the effects in the above studies tend to be highly significant and a mechanistic basis for the effects seem logical [28,29]. Moreover, there is a long history of humans altering their behaviour in response to the detection of infectious disease [55], and such behavioural responses have even been proposed as key drivers of virulence evolution in some systems [56].

In the models presented here, I have followed the standard SIR model assumption that mortality risk is constant for the duration of an infection. This is typically not true [39], with mortality often occurring towards the later phase of infection. If this delay between the start of infection and death were incorporated into my analysis, the effect is that the cost of mortality would be even weaker than I have calculated, further reducing the number of systems in which mortality costs are strong enough to constrain virulence evolution. Notably, a similar delay might occur between infection and detection, such that there is a period of time in which hosts are transmissible but detection is not yet possible. The consequence of this pre-detectable transmission period would be to weaken the cost of detection. An additional point is that mortality costs depend on the infection fatality rate, but for many pathogens, we only have estimates of the case fatality rate (where the demoninator is the number of disease cases) rather than the infection fatality rate (where the denominator is the number of infections). As with nonlinear mortality over time, using case fatality rates would result in overestimating the magnitude of mortality costs.

Note that despite the use of the term ‘detection cost’, my argument is agnostic as to the exact mechanism causing the change in interactions. Multiple mechanisms can result in reduced transmission, and have been documented in human and non-human hosts. Detected infections can result in reduced transmission if infected hosts are too ill to go about their normal routine and thus contact fewer susceptible hosts (e.g. [26]), if they take action to avoid spreading an infection through intentional behavioural modification (e.g. [18,57]), if they seek treatment to end infection earlier (e.g. [58,59]), or even if they are avoided by others who note that they are ill (e.g. [55,56]).

Perhaps the greatest challenge moving forward is to test this theory experimentally. The difficulty of doing so stems from being able to create conditions that are close enough to field conditions such that they allow for changes in behaviour that limit transmission following the detection of infection. Such laboratory experiments may prove too difficult to design, and may ultimately mean that tests of this theory must be performed in the field.

Despite my above argument, there are situations in which a mortality cost can provide a stronger constraint on the evolution of virulence than a detection cost (inequality (2.14); figure 4). For example, mortality costs are extremely large and appear to have been major drivers of pathogen evolution for myxomatosis [51], Marek’s disease virus [11] and some bacteriophages [60]. Notably, these systems tend to have extremely high infection fatality rates, and little opportunity for host behaviour to impact disease dynamics. Likewise, pathogen-induced host mortality plays a major role in the disease dynamics of these systems as evidenced by the fact that mortality effects need to be included to generate accurate models [43,61,62]. Less definitively, capture-mark-recapture studies have demonstrated that some wildlife pathogens (for example, bovine tuberculosis in badgers [63] and chytrid fungus in a rainforest frog [64]) can have infection fatality rates of approximately 30% or higher making them comparable in magnitude to the detection costs seen in the empirical examples described above.

Here, I have assumed that the benefits of virulence come from a correlation with transmission rate (figure 1). This assumption is not critical. As shown by Inequality (2.14), the precise benefit of virulence does not impact whether virulence is more strongly constrained by mortality or detection. Numerous alternative theories have been proposed to explain why pathogens maintain virulence even in cases where virulence itself is not obviously beneficial [3,14,65]. Some of these theories include that multilevel selection leads to the evolution of virulence levels that are non-optimal at the between-host scale (e.g. [66,67]), that spatial structure imposes dispersal or persistence costs of high virulence (e.g. [33]), that environmental feedbacks limit the relationship between the basic reproductive number *R*_0_ and optimal virulence [38], that virulence is not adaptive in the context where it is being studied but adaptive in another context [68], that bottlenecks prevent the evolution of optimal virulence [69], or simply that there is no heritable variation for virulence on which selection can act. My argument is not meant to replace these alternative theories. Each of them is justifiable under certain conditions. Currently, however, these theories are considered as alternatives to a mortality-transmission trade-off. I propose that they should instead be considered as alternatives to a detection–transmission trade-off except in situations where host mortality costs can plausibly be larger in magnitude than detection costs, meaning that either infection fatality rates exceed the 20–80% threshold set by empirical examples of detection costs, or that changes in behaviour are unlikely to alter transmission of that particular system.

While perhaps not obvious, fundamentally different evolutionary conclusions can arise depending on whether costs of virulence are borne out through mortality or detection. For example, imagine a therapeutic drug that is partially effective at reducing transmission and disease severity (perhaps by reducing pathogen loads in hosts). If virulence costs were borne out through mortality, this drug would be expected to drive the pathogen to become more virulent since the pathogen would no longer pay the full mortality cost [11,70]. If virulence costs were instead borne out through detection, this drug could instead drive the pathogen to become less virulent since drug seeking behaviour itself, which is harmful to the fitness of the pathogen, first requires detection. For animals that engage in self-medication behaviour [58], such evolutionary trajectories may be regularly playing out. Assuming mortality and detection are positively correlated, one therefore might expect animal self-medication to cause pathogens with high mortality rates to become more deadly, and to cause pathogens with low mortality rates to become less deadly after controlling for the medication status of the host.

Assuming detection is a main factor in limiting the evolution of virulence, it begs the question of how this knowledge might be used. Ebert & Bull [15] previously argued that virulence management is not practical when it relies on indirect selection using trade-off theory. They instead proposed that efforts would be better aimed towards selecting against virulence directly. I propose that in systems where virulence is constrained by a cost of detection, efforts to increase detection could be quite powerful. In addition to the disease control benefits acquired when infections are detected more often, increased detection could directly select against virulence provided the link between detection and virulence is maintained. Likewise, as we saw during the early days of the COVID-19 pandemic, surveillance programmes are often designed to catch clusters of symptomatic infection [71]. This may unintentionally provide additional evolutionary benefits in that more virulent pathogens will be more likely to be caught and stopped.

A final point is that the precise detection cost paid by pathogens may be due not only to host and pathogen characteristics, but also to exogenous factors. For example, public health policies and diagnostic resources differ among different geographical regions. Likewise, public awareness of infectious diseases can fluctuate over time based on current events, or even differ between individuals in different social networks. The consequences of such exogenous factors may be a particularly rich area for future exploration, including for example, in explaining the maintenance of variation in virulence.

## Data Availability

The data are provided in electronic supplementary material [72].
